# The ENIGI (European Network for the Investigation of Gender Incongruence) Study: Overview of Acquired Endocrine Knowledge and Future Perspectives

**DOI:** 10.3390/jcm11071784

**Published:** 2022-03-24

**Authors:** Carlotta Cocchetti, Alessia Romani, Sarah Collet, Yona Greenman, Thomas Schreiner, Chantal Wiepjes, Martin den Heijer, Guy T’Sjoen, Alessandra Daphne Fisher

**Affiliations:** 1 Andrology, Women’s Endocrinology and Gender Incongruence Unit, Florence University Hospital, Viale Pieraccini 6, 50100 Florence, Italy; carlotta.cocchetti@gmail.com (C.C.); alessiaromani@hotmail.it (A.R.); 2 Department of Endocrinology, Center for Sexology and Gender, Ghent University Hospital, 9000 Ghent, Belgium; sarah.collet@ugent.be (S.C.); guy.tsjoen@ugent.be (G.T.); 3 Institute of Endocrinology and Metabolism, Tel Aviv Sourasky Medical Center, Tel Aviv University, Tel Aviv 6997801, Israel; yonagr@tlvmc.gov.il; 4 Department of Endocrinology, Oslo University Hospital, 0130 Oslo, Norway; thomas@schreiner.no; 5 Department of Endocrinology, Amsterdam University Medical Center, VUmc, 1018 WT Amsterdam, The Netherlands; c.wiepjes@amsterdamumc.nl (C.W.); m.denheijer@amsterdamumc.nl (M.d.H.); 6 Center of Expertise on Gender Dysphoria, Amsterdam University Medical Center, VUmc, 1018 WT Amsterdam, The Netherlands

**Keywords:** gender incongruence, gender dysphoria, transgender, ENIGI, prospective cohort study, gender-affirming hormonal treatment

## Abstract

Literature on the efficacy and safety of gender-affirming hormonal treatment (GAHT) in transgender people is limited. For this reason, in 2010 the European Network for the Investigation of Gender Incongruence (ENIGI) study was born. The aim of this review is to summarize evidence emerging from this prospective multicentric study and to identify future perspectives. GAHT was effective in inducing desired body changes in both trans AMAB and AFAB people (assigned male and female at birth, respectively). Evidence from the ENIGI study confirmed the overall safety of GAHT in the short/mid-term. In trans AMAB people, an increase in prolactin levels was demonstrated, whereas the most common side effects in trans AFAB people were acne development, erythrocytosis, and unfavorable changes in lipid profile. The main future perspectives should include the evaluation of the efficacy and safety of non-standardized hormonal treatment in non-binary trans people. Furthermore, long-term safety data on mortality rates, oncological risk, and cardiovascular, cerebrovascular and thromboembolic events are lacking. With this aim, we decided to extend the observation of the ENIGI study to 10 years in order to study all these aspects in depth and to answer these questions.

## 1. Introduction

Until the 2000s the available literature on the efficacy and safety of gender-affirming hormonal treatment (GAHT) in transgender people was scarce and mostly limited to case reports or small cohort studies. In recent years the number of transgender people seeking GAHT has been progressively increasing. Therefore, the need to move away from case reports and small series has arisen, in order to provide data-driven information on GAHT efficacy and safety. In this context the European Network for the Investigation of Gender Incongruence (ENIGI) collaboration was born. The ENIGI is a prospective, observational, multicentric study, including two branches (ENIGI endocrine and ENIGI mental health protocols). The first participant was enrolled in Ghent, Belgium, in 2010. To date, participating centers in the ENIGI endocrine study have included Ghent, Amsterdam, Florence, Oslo, and Tel Aviv. The multicentric nature of the study has made it possible to reach an important number of enrolled patients, formerly considered to be rare, exceeding at the present moment 2600 inclusions. This has allowed for the production of high-quality data that can be generalized on a larger scale.

The main aims of this collaboration are to describe clinical and side effects of GAHT in transgender people, with a particular focus on metabolic parameters, bone density, physical changes, and psychological well-being. At the present moment, mainly short/mid-term data have been produced.

In this paper, we will review the main evidence that emerged from the ENIGI study since it started 11 years ago, and identify future perspectives and needs. A 10-year follow-up study is timely.

## 2. Description of the ENIGI Study

The ENIGI study is a prospective cohort study. To date, participating gender clinics include Ghent University Hospital, Belgium; VU University Medical Center in Amsterdam, the Netherlands; Florence University Hospital, Italy; Rikshospitalet in Oslo, Norway; and Tel Aviv Sourasky Medical Center, Israel. Study design and methods, as well as the original endocrine protocol, have been extensively described elsewhere [1]. After confirming the presence of gender dysphoria/gender incongruence, patients were included in the ENIGI endocrine protocol at the time of the first hormonal treatment prescription. Included subjects were above 16 years (Oslo), 17 years (Ghent), and 18 years (Amsterdam, Florence and Tel Aviv) of age. Baseline characteristics of the study population enrolled until November 2021 are reported in Table 1.

Enrolled participants were scheduled for an outpatient appointment at baseline (start of GAHT), during the first year (at three, six, and twelve months) and after two and three years. The evaluation included demographic characteristics and clinical history, periodical clinical evaluation and biochemical determinations, as well as bone and body composition measurements [1]. Furthermore, several questionnaires were administered at each time, in order to explore sexual desire, sexual orientation, perceived positive and negative effects, physical activity, aggression proneness, and experienced vocal performance [1] (Table 2). The ENIGI study has continually evolved since its inception. In fact, based on emerging evidence and published data, in 2018 some changes were made to the original ENIGI endocrine protocol, in order to always provide innovative information. More specifically, bioelectrical impedance analysis has been added to the protocol. Also, new questionnaires became part of the assessment in order to evaluate body image perception (Body Image Scale), amenorrhea achievement with testosterone (T) treatment (Menstrual Questionnaire), subjective symptoms (Hormonal Symptoms Questionnaire), reproductive desire (Fertility Desire questionnaire), sleep and mood changes (Pittsburgh Sleep Quality Index; Insomnia Severity Index; Perceived stress scale; Inventory of Depressive Symptomatology Self-Report scale), and ability to regulate emotions (Difficulties in Emotion Regulation Scale) (Table 2).

Regarding GAHT protocols, all participants were treated in line with the World Professional Association for Transgender Health (WPATH) recommendations [2]. Originally, the binary conception of gender was also extended to the transgender experience, limiting hormonal treatment protocols to obtaining a full virilization or feminization/de-virilization. In line with that, trans AMAB (assigned male at birth) people received both anti-androgens and estrogens aimed at obtaining estradiol and testosterone levels within the adult cisgender women’s range. Cyproterone acetate (CPA) is the most commonly used anti-androgen. Initially, CPA was administered at a dosage of 50 mg/daily, which has recently been reduced to 25 mg/daily, and often to 10 mg/daily (range 10–50 mg) in consideration of the similar anti-androgenic effectiveness against a lower risk of side effects [3,4,5]. Among anti-androgenic compounds, alternatives are represented by spironolactone (100–300 mg/daily) and gonadotropin releasing hormone analogues (i.e., triptorelin 3.75 mg per 4 weeks or pamorelin 11.25 mg per 12 weeks). Oral estradiol valerate 2 mg twice a day is the most commonly prescribed estrogenic formulation (2–6 mg/daily). Transdermal estrogens (estradiol patches 100 mcg/24 h twice a week, estradiol hemihydrate gel 1 mg twice daily, estradiol 0.06% gel twice daily) are usually preferred in individuals age > 45 years or with higher thromboembolic risk, due to the by-pass of first-pass hepatic metabolism [1].

Trans AFAB (assigned female at birth) people received—still in a binary perspective of gender—T treatment to obtain full virilization, following the same principles of hormone replacement treatment in hypogonadal cisgender men. Treatment regimens differ between gender clinics due to the variability of reimbursement rules in different European countries. T undecanoate administered intramuscularly (1000 mg per 10–14 weeks with the second injection repeated after 6 weeks) is one of the most frequently prescribed formulations. Other options include T esters intramuscularly (250 mg every 2–4 weeks) or T gel (50 mg daily).

## 3. Materials and Methods

The methodology of this narrative review consisted of a careful analysis of literature on evidence emerging from the ENIGI study regarding efficacy and safety of GAHT. A computerized search was performed independently by two authors using PubMed. Search items included transgender, gender-affirming hormonal treatment, hormonal treatment, ENIGI, European Network for the Investigation of Gender Incongruence, safety, and efficacy. Only original articles were included. Manuscripts emerging from the aforementioned search were selected according with the aims of this review.

## 4. Overview of Acquired Knowledge

### 4.1. Body Modifications Induced by GAHT

#### 4.1.1. Breast Development

GAHT has been shown to be usually effective in inducing body modifications in line with the experienced gender [6]. In trans AMAB people, feminizing hormonal treatment led mostly to a modest increase in breast development after one year, occurring primarily in the first six months [7]. In this study, breast development usually resulted in less than an AAA bra cup size [7]. Even if the maximum effect of estrogens on breast development can be expected after two years [8], the majority of trans AMAB people seek additional breast augmentation besides GAHT [9].

#### 4.1.2. Body Composition and Grip Strength

Using whole body dual-energy X-ray absorptiometry (DEXA), body shape changes in line with experienced gender were observed in both trans AMAB and AFAB people after one year of GAHT [10]. Particularly, estrogen and anti-androgen treatment resulted in increased body fat—especially in the gynoid region—and decreased lean mass, with no differences among different estrogen preparations [10]. On the other hand, T treatment resulted in a decreased percentage of body fat in the gynoid region and increased lean mass [10]. Furthermore, T esters were associated with larger changes in body composition in comparison to testosterone gel [10]. Body mass index (BMI) appeared to be an important determinant of body composition changes, with lower BMI associated with greater modifications [10]. Furthermore, in trans AFAB people, lower T and higher luteinizing hormone levels showed an association with a lack of body composition changes [11].

After one year of GAHT, a decrease in grip strength was observed in trans AMAB people, whereas an increase in trans AFAB people was noted, along with changes in lean body mass [12].

Among physical changes, it seems that GAHT may be able to induce partial modification towards a facial feminization in trans AMAB people and masculinization in trans AFAB people after one year [13].

#### 4.1.3. Dermatological Changes

Considering dermatological outcomes, a small preliminary study demonstrated a relevant growth of facial and body hair after one year of T treatment in trans AFAB people, with a further increase in cases of long-term administration [14]. The presence and severity of acne increased during the first year, with a peak after six months [14].

#### 4.1.4. Vaginal Bleeding

After the start of T treatment in trans AFAB people, the interruption of vaginal bleeding and spotting usually occurs within the first three months [15]. Defreyne and colleagues demonstrated that vaginal bleeding persistence may be associated with lower serum T levels and gel formulation compared to injections (both T esters and undecanoate) [15].

#### 4.1.5. Voice

Finally, GAHT was associated with an improvement in self-perception of voice in both trans AMAB and AFAB people, although only in AFAB people was a directly predictive association with T level increase found [16].

### 4.2. Safety of GAHT

#### 4.2.1. Prolactin Levels

During recent years, the ENIGI study has made a significant contribution to establishing the safety profile of GAHT in transgender people. It is well-known that estrogen treatment induces an increase in prolactin levels. Furthermore, two studies from the ENIGI group highlighted that CPA administration in trans AMAB people may contribute to a mild elevation of serum prolactin with an unknown mechanism, disappearing upon interruption of CPA [17,18].

#### 4.2.2. Erythrocytosis

One of the most common side effects of T treatment in trans AFAB people is represented by erythrocytosis. A large prospective study reported a significant hematocrit increase during T treatment, especially in the first three months [19]. However, serum hematocrit levels were usually in the reference male range and erythrocytosis rates were low in trans AFAB people, with a higher risk in those assuming T esters compared to T undecanoate [19].

#### 4.2.3. Hepatic and Renal Safety

GAHT showed an appropriate hepatic and renal safety profile in the short-term [6]. A recent study found a very low rate of liver injury in transgender people during the first year of GAHT, thus the authors concluded that periodical liver enzyme monitoring would not be necessary [20].

#### 4.2.4. Lipid Profile

Due to the lack of long-term observations, some concerns have been raised about the cardiovascular (CV) safety of GAHT. Regarding CV risk markers, evidence from the ENIGI collaboration demonstrated, in a large population, unfavorable changes in lipid profiles in trans AFAB people and favorable changes in trans AMAB people, even during a mid-term follow-up [21,22]. In particular, in AFAB individuals T treatment led to an increase of total cholesterol, low-density lipoprotein-cholesterol (LDL-c), and triglycerides levels, whereas high-density lipoprotein-cholesterol (HDL-c) levels decreased [21,22]. After the start of GAHT, HDL-c concentrations decreased in both AMAB and AFAB people, however, only in AMAB individuals has a specific reduction in the ATP-binding cassette transporters A1 concentrations been observed, which may compromise the ability of HDL-c to remove cholesterol from arterial wall macrophages, contributing to a higher CV risk [23]. Furthermore, GAHT influences metabolic cytokines levels, such as fibroblast growth factor 21, adiponectin, leptin, chemerin, and resistin [24]. This mechanism may be part of a complex action exerted by sex steroid hormones on several components of metabolic syndrome, including the lipid profile [24].

#### 4.2.5. Insulin Sensitivity

Moreover, GAHT also seemed to affect insulin sensitivity and incretin response. Estrogen plus anti-androgen treatment resulted in a decrease of insulin sensitivity and incretin response to oral glucose load, whereas T treatment showed a positive effect on insulin sensitivity [25].

#### 4.2.6. Coagulation

In trans AMAB people, feminizing hormonal treatment resulted in procoagulant changes, including increased levels of factors IX and XI and decreased levels of protein C [26], which likely contributes to the increased rates of venous thromboembolism (VTE) observed in these individuals [27,28,29].

#### 4.2.7. Cardiovascular Safety

Certainly, all of the aforementioned studies provided relevant insights about GAHT’s effect on the markers of cardiovascular and thromboembolic diseases, and healthy lifestyle seems to be a major contributor. However, data on the real incidence of CV events and VTE are still limited, and only long-term prospective studies will elucidate the potential risks of GAHT in transgender people.

#### 4.2.8. Bone Safety

Since sex steroids are important determinants of bone health, concerns have been raised about a potential impact of GAHT on bone mineral density (BMD). Before the start of GAHT, AMAB trans people were found to have a lower bone mass and higher prevalence of osteoporosis compared to cisgender men, probably due to a less active lifestyle and lower vitamin D levels [30]. In contrast, at baseline, AFAB trans individuals had a similar bone composition compared to cisgender women [30]. When assessing the impact of GAHT through the use of DEXA, an increase in lumbar spine and femoral neck BMD in both AMAB and AFAB people after one year of GAHT was demonstrated [31,32]. Moreover, using peripheral quantitative computed tomography, the preservation of volumetric bone density and geometry was observed in AMAB individuals after two years of GAHT [33]. Changes in bone turnover markers during GAHT confirmed its impact on bone metabolism. Evaluating several bone turnover markers and sclerostin levels, one year of GAHT was associated with lower bone turnover in AMAB people and older AFAB individuals and higher bone turnover in younger AFAB people [34]. This was probably due to the fact that the older group of AFAB people benefited the most from GAHT, as they were assumed to have lower estrogen levels at baseline [34]. No significant changes in vitamin D status were found during GAHT [35].

#### 4.2.9. Subjective Changes during GAHT

The perceived efficacy and tolerability of GAHT were assessed prospectively through a structured questionnaire in the ENIGI protocol [36]. During a 12-month follow-up, trans AFAB people persistently reported hot flashes, balding, voice instability, acne, and increase in sexual desire, whereas trans AMAB people reported hot flashes, night sweats, fatigue, weight gain, breast tenderness, emotional instability, and mood swings [36].

#### 4.2.10. Emotional, Psychological, and Sexual Related Aspects

So far, the ENIGI collaboration has not just focused on the purely medical aspects of GAHT, but also explored several features of psychological and sexual well-being. This research field plays a key role, as the quality of life of transgender people represents one of the most important goals of gender-affirming care. When assessing changes in positive and negative emotional states and attitudes during gender-affirming paths, a decrease of both was found during the first three months of GAHT, probably related to the social difficulties experienced [37].

Since some guidelines warn about aggression in trans AFAB people after the start of T treatment, the ENIGI group evaluated anger intensity modifications on a large sample during a three-year follow-up [38]. No significant changes in anger intensity were found either in trans AMAB or AFAB people after the start of GAHT, and no association between state-level anger intensity and serum T levels emerged [38].

Regarding sexual well-being, it is known that sexual distress decreases in both trans AFAB and AMAB people during GAHT administration, probably in relation to the improvement of body image perception [39]. Furthermore, GAHT seemed to affect sexual desire in the short-term, with a significant decrease in trans AMAB people in the first three months, probably due to anti-androgen administration [40]. However, over a longer period of time a net increase in sexual desire was observed in trans AMAB individuals, whereas in the long-term no relevant changes were found in trans AFAB people with respect to baseline [40]. Moreover, sexual orientation did not change over time after the start of GAHT [41].

Data on GAHT safety emerging from the ENIGI study are summarized in Table 3.

## 5. Future Perspectives and Conclusions

Despite growing evidence based on data from many participants, there are still many questions that need to be answered. First of all, to date the ENIGI collaboration has evaluated only the efficacy and safety profile of standardized GAHT in trans people requesting full de-/masculinization or de-/feminization. However, non-binary transgender people represent a growing body of those who refer to gender clinics, and requests for non-standardized GAHT are increasing [42,43]. In line with this, some authors hypothesized both pharmacological and non-pharmacological strategies to respond to the different requests of non-binary people [44,45]. Given the complete lack of data on the efficacy and safety of these treatments, the ENIGI collaboration should focus more on this issue in the near future, and given the large number of participants, individualized variations in hormone treatment are now welcomed.

Moreover, little is known regarding the safety of GAHT in elderly transgender people. This includes both the possible reduction/discontinuation of GAHT in elderly individuals and the start of GAHT later in life. In fact, to date, recommendations for the endocrine management of this population reflect the experience based on sex hormone replacement treatment in cisgender aging people [46]. This represents another aspect to focus on, in order to make evidence-based recommendations in the future.

Aiming to produce the most rigorous evidence possible, until now the ENIGI study has focused above all on objective and measurable changes induced by GAHT. In the future, it will be important to integrate these data with those subjectively reported by transgender people, evaluating changes in body image perception, psychological well-being, and quality of life after the start of GAHT.

One of the main shortcomings of scientific literature concerns the data regarding the long-term safety of GAHT. Producing data on mortality rates, oncological risk and incidence of cardiovascular, cerebrovascular and thromboembolic events should be the main prerogative of future research. Furthermore, although GAHT did not appear to be associated in the short/mid-term with BMD impairment, evidence related to long-term fracture risk is limited and needs to be implemented [47]. With this aim, we decided to extend the observation of the ENIGI study to 10 years in order to study all of these aspects in depth and to answer these questions. Because of an increasing group of people requesting GAHT, and based on reassuring shorter time data, we have chosen to also investigate new participants at larger intervals (baseline, 3 months, 12, 24 and 36 months). Randomized controlled trials of different hormone regimens are also possible, and more translational research may support and elucidate earlier findings.

To conclude, transgender medicine research has finally evolved from small studies and case reports. Today this research field consists of large prospective studies (including the ENIGI collaboration), which until now produced evidence mainly at short/mid-term. Even if this evidence is reassuring, it is time to take the investigations further, providing long-term data.

## Figures and Tables

**Table 1:** Baseline characteristics of the study population.

	AMAB (N = 1261)	AFAB (N = 1411)	Total (N = 2672)
Study center (%)			
Amsterdam	810 (64.2%)	873 (61.9%)	1683
Ghent	345 (27.4%)	296 (21.0%)	641
Oslo	30 (2.4%)	141 (10%)	171
Florence	67 (5.3%)	90 (6.3%)	157
Tel Aviv	9 (0.7%)	11 (0.8%)	20
Age (years)	26.6 (22.0–38.8)	22.3 (19.9–27.3)	23.9 (20.6–32.4)
Current smokers N (%)	22.5%	28.6%	25.7%
Weight (Kg)	72.0 (63.5–83.5)	67.0 (58.0–80.0)	69.0 (60.1–82.0)
Height (m)	1.78 ± 0.07	1.67 ± 0.07	1.72 ± 0.09
BMI (Kg/m^2^)	22.6 (20.1–25.9)	23.7 (21.0–28.6)	23.2 (20.6–27.1)
Systolic blood pressure (mmHg)	127.0 ± 15.0	120.0 ±13.0	123.0 ± 14.0
Diastolic blood pressure (mmHg)	78.0 ± 10.0	75.0 ± 9.0	77.0 ± 10.0

Data are shown as median with interquartile range for non-normally distributed data and mean ± standard deviation for normally distributed data. Reported data refer to inclusions until the end of November 2021. AMAB = assigned male at birth, AFAB = assigned female at birth, BMI = body mass index.

**Table 2:** Details of original and new ENIGI endocrine study protocol.

Original Study Protocol	
Clinical evaluation	Height, weight, BMI, blood pressure, circumferences (waist, hip, chest, breast), grip strength, Norwood Hamilton scale for androgenetic alopecia, Ferriman Gallwey score for body and facial hair distribution, GAGS for acne activity.
Laboratory measurements	17-beta estradiol, testosterone, SHBG, LH, FSH, prolactin, IGF-1, blood count, calcium, vitamin D, albumin, TSH, alkaline phosphatase, gamma-GT, AST/ALT, triglycerides, total cholesterol, HDL-c, LDL-c, glucose, insulin, creatinine.
Imaging	Dual-energy X-ray absorptiometry.
Questionnaires	Sociodemographic and clinical information
	Baecke Activity Questionnaire This questionnaire allows the assessment of habitual physical activity. It consists of 16 questions classified into 3 different dimensions: work, sport, and non-sports leisure activity. Each domain can receive a score from 1 to 5 points, thus allowing a total score from 3 (minimum activity) to 15 (maximum activity).
	Positive and Negative Affect Scale (PANAS) The PANAS is a 20-item questionnaire that measures long-term changes in affect. Participants are asked to what extent they experience certain feelings such as anxiety, happiness, or guilt on a 5-point scale from “very little” to “very much”.
	State Anger Scale The State Anger scale is a 15-item questionnaire that evaluates aggression. Participants rank certain statements along a 4-point continuum from “not at all” to “very much”. The questionnaire evaluates angry feelings on 3 subscales: feeling angry, feeling like expressing anger verbally, and feeling like expressing anger physically.
	Sexual Desire Inventory (SDI) SDI is a self-administered 14-item questionnaire that aims to measure sexual desire. The SDI measures the individual’s thoughts as well as actual experiences. Fourteen questions assess the strength, frequency, and importance of an individual’s desire for sexual activity with others and by themselves. The score ranges from 0 (no sexual desire) to 112 (maximum desire).
	Sexual orientation questionnaire (SEXOR) This short questionnaire consists of 4 questions in the background data interview evaluating gender roles in sexual fantasy and sexual orientation.
	Trans Voice Questionnaire The Trans Voice Questionnaire is a 30-item self-administered questionnaire that evaluates the psychosocial consequences of voice disorders and consists of 3 dimensions: functional, physical, and emotional impairment. A score of 0 is equivalent to no disability and a score of 120 is equivalent to maximum disability.
	Side-effects questionnaire This questionnaire evaluates side-effects of hormonal treatment such as psychovegetative symptoms, physical complaints, cognition, emotionality and sexuality, genital complaints, and pain.
New study protocol	
Clinical evaluation	Height, weight, BMI, blood pressure, heart rate, BIA.
Laboratory measurements	17-beta estradiol, testosterone, LH, blood count, AST/ALT, triglycerides, total cholesterol, HDL-c, LDL-c, glucose, creatinine.
Questionnaires	Sociodemographic and clinical information
	Baecke Activity Questionnaire
	Body Image Scale (BIS) The BIS questionnaire consists of 30 body characteristics. Respondents rate satisfaction with these body characteristics on a 5-point scale, ranging from 1 (very satisfied) to 5 (very dissatisfied). There are two sex-specific versions of the BIS—one for natal males and one for natal females—containing equivalent genital body parts. The scale includes primary sex characteristics, secondary sex characteristics, and non sex-related body parts. Higher scores represent higher degrees of body dissatisfaction.
	Menstrual questionnaire This questionnaire evaluates presence and characteristics of menstrual cycle, in order to establish amenorrhea achievement with testosterone treatment in trans AFAB people.
	Hormonal symptoms questionnaire This short questionnaire assesses the presence and intensity of several perceived symptoms, such as psychoneurovegetative symptoms, physical complaints, emotionality and sexuality complaints.
	Fertility questionnaire This questionnaire contains several questions, in order to assess reproductive desire of transgender people.
	Pittsburgh Sleep Quality Index (PSQI) PSQI is a self-rated questionnaire which assesses sleep quality and disturbances over a one-month time interval. Nineteen individual items generate seven “component” scores: subjective sleep quality, sleep latency, sleep duration, habitual sleep efficiency, sleep disturbances, use of sleeping medication, and daytime dysfunction. The sum of scores for these seven components yields one global score.
	Insomnia Severity Index (ISI) ISI is composed of seven items evaluating: (a) the severity of sleep-onset (initial), (b) sleep maintenance (middle), (c) early morning awakening (terminal) problems, (d) satisfaction with current sleep pattern, (e) interference with daily functioning, (f) noticeability of impairment attributed to the sleep problem, and (g) level of distress caused by the sleep problem. Each of these items is rated on a five-point Likert scale (‘0’ = not at all, ‘4’ = extremely) and the time interval is in the last two weeks. Total scores range from 0 to 28, with high scores indicating greater insomnia severity.
	Perceived stress scale (PSS) The PSS is an instrument used to measure stress, evaluating the perception of stressful experiences in the preceding month using a Likert-type five-point scale. PSS contains 14 items, seven of them positive and seven negative, and responses range from 0 to 4 (0 = never; 1 = almost never; 2 = sometimes; 3 = fairly often; 4 = very often).
	Inventory of Depressive Symptomatology Self-Report scale This scale assess the presence and intensity of depressive symptoms. Items are scored on a four-point scale, with each item equally weighted and summed to a total score. A higher total score indicates more serious depression, with a maximum score of 84.
	Difficulties in emotion regulation scale (DERS) DERS is a 36-item scale assessing difficulties in six regulatory abilities: lack of emotionalawareness, lack of emotional clarity, non-acceptance of emotional responses, difficulties engaging in goal-directed behavior, impulse control difficulties, and limited access to emotion regulation strategies. The scale is a Likert-type scale rated between 1 (almost never) and 5 (almost always) points, with higher scores indicating more regulation difficulties.

BMI = body mass index; GAGS = Global Acne Grading system; SHBG = sex hormone binding globulin; LH = luteinizing hormone; FSH = follicle stimulating hormone; IGF1 = insulin-like growth factor 1; AST = aspartate aminotransferase; ALT = alanine aminotransferase; HDL = high-density lipoprotein; LDL = low-density lipoprotein; BIA = bioelectrical impedance analysis.

**Table 3:** Data on GAHT safety emerging from the ENIGI study.

	AMAB	AFAB
Biochemical monitoring	▪ Appropriate hepatic and renal safety in the short-mid term ▪ Increase of prolactin levels during estrogen plus CPA administration	▪ Appropriate hepatic and renal safety in the short-mid term ▪ Significant hematocrit increase, especially in the first three months, with serum hematocrit levels usually in the reference male range
Cardiovascular safety	▪ Decrease of total cholesterol, LDL-c, HDL-c and triglycerides concentrations ▪ Reduction of ATP-binding cassette transporters A1 concentrations which may influence CV risk ▪ Metabolic cytokines changes (FGF-21 increase, resistin decrease), which may explain some changes in different components of the metabolic syndrome ▪ Procoagulant modifications (increased levels of factor IX, XI and decreased levels of protein C)	▪ Unfavorable lipid changes with an increase of total cholesterol, LDL-c and triglycerides levels and decrease of HDL-c levels ▪ Metabolic cytokines changes (FGF-21 and adiponectin decrease), which may explain some changes in different components of the metabolic syndrome ▪ Increase of 30-years CV risk assessed though the Framingham estimate
Bone safety	▪ Increase in lumbar spine and femoral neck BMD in the mid-term ▪ Preservation of volumetric bone density and geometry ▪ Reduction of bone turnover markers levels	▪ Increase in lumbar spine and femoral neck BMD in the mid-term ▪ Reduction of bone turnover markers levels only in younger individuals (aged <50 years)
Emotional aspects and sexual health	▪ Decrease in sexual desire in the first three months ▪ Decrease of perceived sexual distress in the mid-term ▪ No changes in sexual orientation	▪ No significant changes in anger intensity ▪ Increase in sexual desire in the first three months ▪ Decrease of perceived sexual distress in the mid-term ▪ No changes in sexual orientation

AMAB = assigned male at birth; AFAB = assigned female at birth; CPA = cyproterone acetate; HDL = high-density lipoprotein; LDL = low-density lipoprotein; CV = cardiovascular; FGF = fibroblast growth factor; BMD = bone mineral density.

## Data Availability

Not applicable.
